# Anti-diabetes effect of water containing hydrogen molecule and Pt nanoparticles

**DOI:** 10.1186/1753-6561-5-S8-P18

**Published:** 2011-11-22

**Authors:** Sanetaka Shirahata, Takeki Hamasaki, Keisuke Haramaki, Takuro Nakamura, Masumi Abe, Hanxu Yan, Tomoya Kinjo, Noboru Nakamichi, Shigeru Kabayama, Kiichiro Teruya

**Affiliations:** 1 Department of Bioscience and Biotechnology, Faculty of Agriculture, Kyushu University, 6-10-1 Hakozaki, Higashi-ku, Fukuoka 812-8581, Japan; 2 Division of Life Engineering, Graduate School of Systems Life Sciences, Kyushu University, 6-10-1 Hakozaki, Higashi-ku, Fukuoka 812-8581, Japan; 3 Nihon Trim Co. Ltd., 1-8-34 Oyodonaka, Kita-ku, Osaka 531-0076, Japan

## Background

Electrochemically reduced water (ERW) contains a lot of hydrogen molecule (H_2_) and scavenges reactive oxygen species (ROS) to protect DNA from oxidative damage [1]. ERW also contains small amounts of Pt nanoparticles (NPs) and elongates the lifespan of *C. elegans *[2]. Pt NPs are newly recognized multi-functional ROS scavengers [3]. ERW exhibits anti-diabetes effects *in vitro* and *in vivo *[4-6][7]. We proposed mineral nanoparticle active hydrogen reduced water hypothesis to explain the activation mechanism of H_2_ to hydrogen atom (H)[4]. Recently, H_2_ has been reported to scavenge ROS and suppress a variety of oxidative stress-related diseases [8], however, the action mechanism of H_2_ has not been clarified thoroughly. Here, we examined anti-diabetes effects of H_2_ and Pt NPs.

## Materials and methods

Pt NPs of 2-3 nm sizes were synthesized from H_2_PtCl_6_ by the citrate reduction method. L6 rat myoblast cells (1.2 x 10^5^ cells) were inoculated into a 35 mm culture dish and a day later, the cells were treated with or without 25mM N-acetylcystein in the presence of BES-H2O2, a H_2_O_2_-specific detection reagent in DMEM for 2 h. After washing the cells, molecular hydrogen treatment was performed in a dark condition by cultivating cells in a fresh DMEM medium in a mixed gas incubator under an atmosphere of 75%N_2_/20%O_2_/5%CO_2_ or 75%(H_2_ and N_2_ mixed gas)/20%O_2_/5%CO_2_ for 1.5 h, followed by flowcytometric analysis. In this condition, culture medium contained maximum 0.4-0.5 ppm of dissolved hydrogen. Glucose uptake of differentiated myotube L6 cells was examined after treating the cells with ^3^H-2-deoxyglucose for 10 min. Gene expression of catalase (CAT), glutathione peroxidase (GPx) and hemoxoigenase (HO-1) was examined using RT-PCR method. Three weeks old type 2 diabetes model mice (KK-*A^y^*) were fed H_2_ and/or Pt Nps-containing water *ad lib* for 6 weeks.

## Results

**Figure 1:**
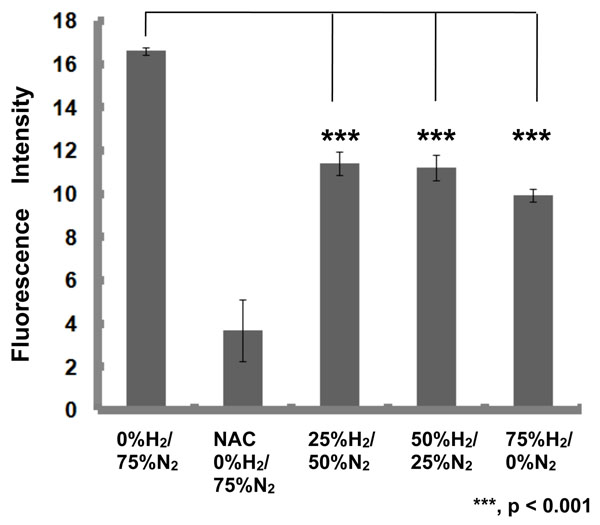
The scavenging effect of hydrogen molecule on intracellular hydrogen peroxide in rat myotube L6 cells. ***, p<0.001.

**Figure 2:**
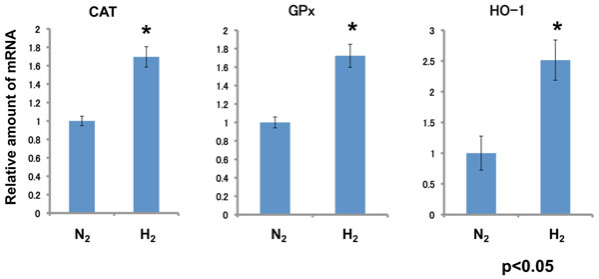
Induced gene expression of anitoxidative enzymes by hydrogen molecule. L6 myoblast cells were cultivated under an atmosphere of 75%N_2_ or H_2_/20%O_2_/5%CO_2_ for 2 h and gene expression was analyzed by RT-PCR. *, P<0.05

H_2_ stimulated glucose uptake into L6 cells. Pt NPs catalyzed the activation of H_2_ to hydrogen atom (H) to scavenge DPPH radical *in vitro*. The combined use of molecular hydrogen and Pt NPs resulted in extremely stimulated glucose uptake into L6 cells, suggesting that H produced from H_2_ by catalyst action of Pt NPs regulated glucose uptake signal transduction. As oppose to the paper by Ohsawa et al.[8], H_2_ of 25 to 75% concentration in the mixed gas significantly scavenged intracellular H_2_O_2_ in rat fibroblast L6 cells (Figure 1) and induced the gene expression of antioxidative enzymes such as CAT, GPx and HO-1 via activation of Nrf2 (Figure 2). H_2_, Pt NPs and their combination significantly suppressed the levels of fasting blood glucose and improved the impaired sugar tolerance abilities of obese insulin-resistant type 2 diabetic KK-*A^y^* mice.

## Conclusion

H_2_, Pt NPs, and their combined use resulted in activation of glucose uptake signal transduction pathways and stimulation of glucose uptake into L6 myotubes. In the groups of H_2_, Pt NPs and their combined use groups, blood sugar levels and impaired sugar tolerance of type 2 diabetes model mouse (KK-*A^y^*) were significantly improved, suggesting that H_2_, Pt NPs and H are redox regulation factors in animal cells.
